# Increased Insensible Water Loss Contributes to Aging Related Dehydration

**DOI:** 10.1371/journal.pone.0020691

**Published:** 2011-05-31

**Authors:** Natalia I. Dmitrieva, Maurice B. Burg

**Affiliations:** Laboratory of Kidney and Electrolyte Metabolism, National Heart, Lung, and Blood Institute, National Institutes of Health, Bethesda, Maryland, United States of America; INSERM, France

## Abstract

Dehydration with aging is attributed to decreased urine concentrating ability and thirst. We further investigated by comparing urine concentration and water balance in 3, 18 and 27 month old mice, consuming equal amounts of water. During water restriction, 3 month old mice concentrate their urine sufficiently to maintain water balance (stable weight). 18 month old mice concentrate their urine as well, but still lose weight (negative water balance). 27 month old mice do not concentrate their urine as well and lose even more weight than the 18 month old mice, indicating a larger negative water balance. Negative water balance in older mice is accompanied by increased vasopressin excretion, providing further evidence of dehydration. All 3 groups maintain water balance while consuming only the water in gel food containing 56% water. However, both older groups excrete a smaller volume of urine of higher osmolality, indicating greater extra urinary water loss. Since their feces also contain less water, the excess water lost by the older mice apparently is through other routes, presumably insensible loss through the respiratory tract and skin. The greater insensible water loss occurs at an earlier age (18 months) than decreased urine concentrating ability (27 months). We propose that insensible water loss through skin and respiration increases with age, making a major contribution to aging related dehydration.

## Introduction

Water balance equates water input and output over time. The major route of intake of water is by ingestion of fluids and food. Food contains water, and additional water is produced during oxidation of carbohydrates. The major routes of water loss are urine, feces, sweat and insensible water loss by evaporation from the respiratory tract and diffusion through the skin [1].

Aging is associated with a tendency to negative water balance, making older subjects more prone to dehydration. Studies of the aging related disturbance of water balance have mostly focused on urinary concentrating ability. The maximum concentration of the urine generally decreases with age [2], [3]. The aging related reduction of urine concentrating ability was attributed to decreased sensitivity to the antidiuretic hormone, vasopressin (AVP) [4], [5]. Renal medullary function is essential for concentrating the urine. Many of the key transport proteins that contribute to urine concentrating ability are reduced in the medulla of aged rats (reviewed in [6]). The basis for this is incompletely understood. We previously found that cellular senescence is accelerated in renal medullary cells, which is attributable to the high interstitial NaCl concentration in that tissue, and we proposed that cellular senescence is an important factor in the aging related decrease of urine concentrating ability [7]. An additional factor that contributes to dehydration with aging is inadequate water intake because of inappropriately decreased thirst [8].

Insensible water loss is a major component of water balance, comparable in daily volume to the urine. In humans the average water loss by diffusion through the skin is about 300 to 400 ml/day, and an approximately equal amount is lost through the respiratory tract. The combined total of 600–800 ml/day represents 30–50% of all water loss, depending on the level of water intake [1]. Thus, insensible water loss is an important component of water balance, and could be decisive when water intake is low.

We previously suggested that extra renal water loss might increase with age [9]. Ku86−/− mice age prematurely [10], [11], including premature senescence of cells in their renal inner medullas [9]. We compared urinary concentrating ability of young (3 month old) Ku86−/− mice to young and old (14–24 month old) wild type mice. Upon water restriction, urine osmolality is lower in both young Ku86−/− and old wild type mice than in young wild type mice, as might be expected from the premature aging of Ku86−/− mouse kidneys. Of note, during water restriction, the lower osmolality of the urine from the old mice was not accompanied by a proportionate increase in volume. We hypothesized that insensible water loss might increase with age, contributing to disturbed water balance [9]. In the present studies we directly tested that possibility by measuring water balance, including water content of feces and urine, during graded water restriction. We find that insensible water loss increases with age in mice, making a major contribution to the aging related deficit of water balance, and that the increased insensible water loss precedes the diminished urinary urine concentrating ability.

## Results

### Mice of different ages all consume the same amount of gel food regardless of its water content

**Figure 1:**
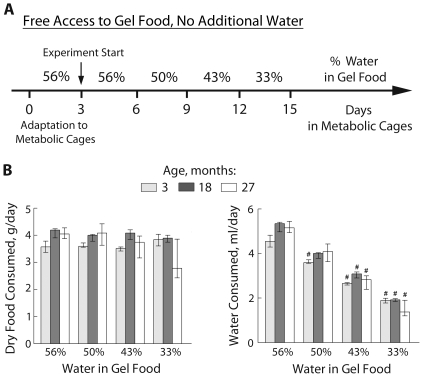
Setup to measure water balance in mice of different ages. A) Diagram showing experimental design. Mice were placed in metabolic cages and subjected to 4 consecutive periods (3 days each) of progressively reduced water content (from 56% to 33%) of their gel food. B) Consumption of food and water, calculated based on the water content of the gel food and the amount of gel food consumed each day (median and IQR, n = 3–4, ^#^ P<0.05 relative to 56% water in gel food). 3 and 18 month old mice eat similar amount of food at all water availability that does not change with decreasing water content. 27 month old mice decrease amount of food eaten when water content decreases to 33%.

Figure 1B shows the daily consumption of food and water, combined in a gel. Reducing the water content of the gel food decreases water intake, but intake of food remains constant. Although the older mice are larger, the amount of food that they consume is similar to that consumed by the younger mice over the course of the experiment. The exception is that 27 month old mice consumed less during the last period when water content of the gel food was decreased to 33%. Those mice were very dehydrated by then (see below). Since water intake was similar for the mice of different ages, any differences in hydration were due to differences in water loss.

### Effect of decreased water intake on hydration of mice of different ages

**Figure 2:**
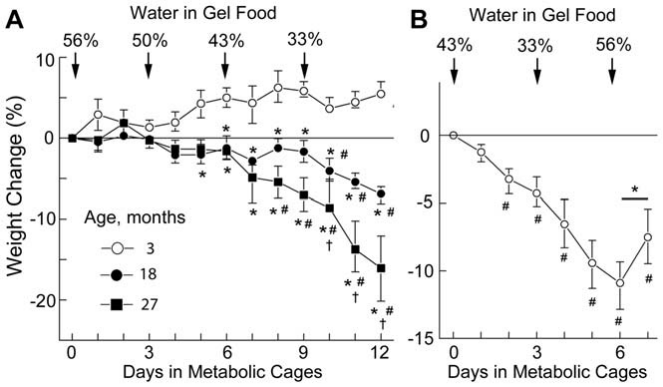
Analysis of hydration based on changes of body weight. A) Changes of body weight resulting from decreased water intake in mice of different ages. Data are presented as % weight change relative to the weight at the beginning of the experiment (median and IQR, n = 3–4, * P<0.05 relative to 3 month old mice; # P<0.05 relative to 0 time; ^†^ P<0.05 relative to 18 month old mice). 3 month old mice do not lose weight, despite decreasing water intake. 18 and 27 month old mice lose weight when water content of the gel food falls to 43% - 33%. 27 month old mice lose the most weight and are very dehydrated by the end of the experiment. B) Reversal of progressive weight loss when low water content of the gel food is increased. 16 month old mice were subjected to consecutive periods (3 days each) of 43%, then 33% water in their gel food, which caused weight loss that progressed over 6 days. On day 7 when the water content of the gel food was increased to 56%, the mice began regaining weight. This rapid onset of weight gain when water intake was increased supports our use of weight as a measure of water balance. Data are plotted as Median and IQR; n = 4, (^#^) P<0.05 relative to 0 time. (*): P = 0.06 if Mann-Whitney Test (one-tailed, paired) is used, P = 0.002 if Student t-test (one-tailed, paired) is used. Note: the experiments shown on panels A and B are not directly compatible because the diets differed (see Methods for details).

Body weight is a simple and accurate index of hydration when serial measurements are made in close proximity [12]. As long as water input and output are balanced, body weight stays constant. Acute dehydration is reflected by an immediate decrease in body weight. Figure 2A shows the change in body weight of mice of different ages as their water intake is decreased. When their gel food contains 56% water, mice of all ages maintain water balance, as indicated by constant weight. With reduced water intake 3 months old mice maintain water balance, as indicated by a near constant weight (that initially even increases slightly). In contrast, 18 and 27 month old mice become dehydrated (lose weight) when there is less than 50% water in their gel food, and dehydration becomes progressively severe as water intake falls further. The dehydration is greater in 27 month old than 18 month old mice. Fast recovery of weight after water intake increased supports the conclusion that the weight loss during water restriction was caused by dehydration (Fig. 2B).

### Effect of age on water loss by different routes

**Figure 3:**
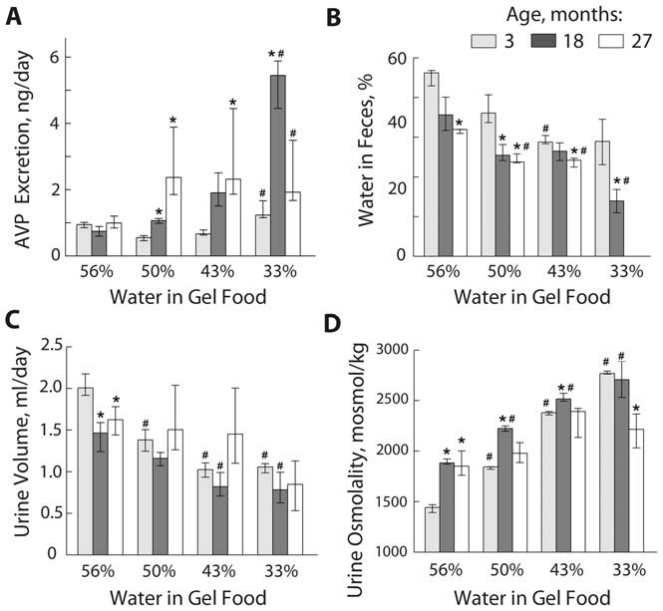
Analysis of activation of water conservation mechanisms at different levels of water consumption. A) AVP excretion; B) % of water in feces; Note: data are missing for 27 month old mice when gel food contained 33% of water because the mice became constipated; C) Urine volume; D) Urine Osmolality. Data are presented as median and IQR, n = 3–4, * P<0.05 relative to 3 months old; ^#^ P<0.05 relative to 56% water in gel food; Mann-Whitney Test (one-tailed).

Mice of all ages maintained water balance (stable weight) when their gel food contained 56% water (Fig. 2). Consistent with ample hydration, all had low levels of AVP excretion (Fig. 3A). Despite similar water intake (Fig. 1B), however, 18 and 27 month old mice excreted less urine (Fig. 3C) of higher osmolality (Fig. 3D) and had less water in their feces (Fig. 3B) than 3 month old mice. The most plausible explanation for this difference is greater insensible water loss by the older mice. When the water content of their gel food was decreased (Fig. 1B), 3 month old mice adjusted by excreting less urine (Fig. 3C) of higher osmolality (Fig. 3D), and they remained in water balance, as evidenced by stable weight (Fig. 2). 18 month old mice concentrated their urine as much or more than 3 month old mice (Fig. 3C and 3D) and excreted less water in their feces (Fig. 3B), yet they lost weight (Fig. 2), confirming greater insensible water loss than 3 month old mice. 27 month old mice did not concentrate their urine, decrease urine volume (Fig. 3C), or increase their urine osmolality as much as younger mice (Fig. 3D), indicating that their greater dehydration during water restriction (Fig. 2) involved both insensible water loss and loss of water in urine because of decreased concentrating ability. Note that reduced urine volume in 27 month old mice when their gel food contained 33% of water (Fig. 3C) was not due to increased urine concentration (Fig. 3D). We conclude that insensible water loss increases with age in mice and that this occurs at an earlier age (18 months) than decreased urine concentrating ability (27 months).

### AVP excretion

Negative water balance increases AVP, which stimulates water conservation by the kidneys and colon. We analyzed the effects of water intake, age and hydration on urinary AVP excretion (Fig. 3A). Mice of all ages excreted similar amounts of AVP while ingesting gel food containing 56% water and they maintained water balance. 3 month old mice remained in water balance throughout the experiment, despite water restriction, by decreasing their urine volume (Fig. 3C) and increasing urine osmolality (Fig. 3D) with little measurable change in AVP excretion (Fig. 2A). In contrast, 18 and 27 month old mice greatly increased their AVP excretion (Fig. 3A and 2).

## Discussion

We analyzed mice of different ages to investigate the factors that contribute to the disturbance of water balance with aging, and found that insensible water loss increases with age, contributing to the dehydration that occurs when water intake is reduced. The two major routes of insensible water loss are diffusion through skin and evaporation from the respiratory tract, but the effects of aging on them have not been extensively studied.

One route of insensible water loss is passive diffusion of water vapor through the barrier of the skin (Trans-Epidermal Water Loss, TEWL). Instrumentation developed to measure TEWL includes both open and closed chamber devices [13], [14]. Although deterioration of the skin with age increases its susceptibility to injury and slows repair of its barrier function, basal TEWL was found to be unchanged, or even decreased, with aging both in humans and mice ([15] and reviewed in [16]). Nevertheless, older mice are larger, so there could be more evaporation through the greater area of skin. While the mice were in water balance, eating gel food with 56% water content, the older mice excreted 42±5% less urine, while their calculated body surface area is 25±5% greater. Considering that the older mice also had less water in their feces, the greater skin surface area, without any change in water permeability of the skin, apparently accounts for only part of the difference in insensible water loss.

Increased respiratory evaporation could also contribute to increased insensible water loss during aging. Aged normal lungs have dilated alveoli, enlarged airspaces, decreased exchange surface area and loss of supporting tissue in the peripheral airways [17], [18]. However, in the absence of direct measurements of respiratory water evaporation we cannot evaluate its possible contribution to aging related dehydration. These limitations call attention to the need for additional studies using novel methods if we are to understand the basis for the age related increase of insensible water loss.

We found that the older mice, but not the youngest ones, increased excretion of AVP in their urine when their intake of water was reduced (Figure 3A). Despite numerous studies of the effect of aging on plasma AVP in various animal species, including humans, it has still is not been clear how aging affects the level of AVP. The results are conflicting. While some studies report increased basal plasma AVP with aging, others do not, and some even report a decrease (reviewed in [19]). In our experiments excretion of AVP was equal in mice of all ages while they were in water balance, consuming equal amounts of gel food containing 56% water (Figure 3A). However, when water was restricted, the older, but not youngest, mice increased their excretion of AVP, as they went into negative water balance due to increased insensible water loss. Thus, differences in hydration might have contributed to variability of the findings concerning the effect of aging on the level of AVP.

Summary and conclusions: 1) While consuming gel food containing 56% water, mice of all ages consume the same amount of food and water and maintain water balance, but the older mice excrete less urine of higher osmolality and excrete drier feces (Fig. 3). Thus, older mice have increased insensible water loss. 2) When water is restricted, older mice compensate the increased insensible water loss by increasing AVP excretion, accompanied by further concentration of their urine and lower water content of their feces. 3) The higher AVP that maintains hydration in older mice could contribute to age related pathology [20], [21]. 4) Young mice require less water to maintain hydration (Figure 2). 5) Urine concentrating ability of 27 month old mice is less than that of the 3 and 18 month old mice, as seen when water intake is lowest (Figure 3D). 6) Increased insensible water loss occurs at a younger age (18 months) than decrease of urine concentrating ability (27 months).

## Materials and Methods

### Ethics Statement

All mouse studies were done under Protocol H-0130 approved by Animal Care and Use Committee (ACUC) of National Heart, Lung, and Blood Institute (NHLBI).

### General considerations about experiment design

To analyze extra-renal water loss we designed our experiment so that water and food consumption by mice of different ages was similar with stages of graded water intake. We started with enough water to maintain water balance (stable weight), then gradually decreased water intake to activate its conservation and assay urine concentrating ability. We previously found [9] that when the sole source of water is gel food containing 43% or less water, old mice began to lose weight. Therefore, in present experiment we started with gel food containing more water, namely 50% and 56%. We found that, given free access to unlimited amount of such food, mice eat the same amount of food (dry weight), so they get different amounts of water, depending on how much water is added to the food.

### Measuring water balance

Wild type mice were purchased at age of 2 months from Taconic (129S6, Model no. 129SVE, Taconic Farms, Inc, Hudson, NY) and housed in Association for Assessment and Accreditation of Laboratory Animal Care-accredited facilities of NHLBI. During housing at the Animal facility, mice used for main experiment were fed with Mouse Diet 5021 (LabDiet, Richmond, Indiana). Mice used for experiment described on Fig. 2B were fed with balanced purified rodent diet (AIN-76A, Research Diets, New Brunswick, NJ).

Water balance was assayed by weighing the mice [22], [23]. During the experiment, mice were housed in metabolic cages (Hatteras Instruments, Cary, NC) with controlled temperature and light (12-h light and dark cycles). There were 3 groups of mice of different ages: 1) 3 months old (4 mice, weight = 25.3±0.6 g, mean± SEM): 2) 18 months old (4 mice, weight = 37.2±2.9 g); 27 months old (3 mice, weight = 36.8±3.7). The experiment design is shown in Figure 1A. Initially, all mice received gelled food containing 56% water: 5 ml of deionized water+4 g of balanced purified rodent diet (AIN-76A, Research Diets, New Brunswick, NJ) +90 mg of agar per 9 g of the food. Food was provided in excess in individual cups so the mice ate what they wanted. After 3 days of adaptation to the metabolic cages and the gel food containing 56% of water, mice were subjected to 4 consecutive periods (3 days each) during which they received gel food with progressively lower water content: 56%, 50% (4 ml of water+4 g of the rodent diet powder+80 mg of agar), 43% (3 ml of water+4 g of the rodent diet powder+70 mg of agar) and 33% (2 ml of water+4 g of the rodent diet powder+60 mg of agar). Body weight, urine volume, food consumption and urine osmolality were measured every 24 h. AVP excretion in urine and the water content of feces were measured at the end of each period. Urine was collected under mineral oil in pre-weighed collection vials. The volume was measured gravimetrically, assuming a density of one. Gel food was supplied in pre-weighed plastic cups to facilitate measurement of food consumption. Urine osmolality was measured with a Fiske Model 210 Freezing-Point Micro-Osmometer (Fiske Associates, Norwood, MA). AVP concentration in urine was measured using Vasopressin Enzyme Immunoassay Kit (no. 900-017, Assay Designs, Ann Arbor, MI). Water content of feces was measured by collecting feces in pre-weighed tubes, while the mice were being weighed, and weighing the tubes before and after drying it in chemical hood for 3 days.

#### Statistical Analysis

Data are presented as median and interquartile range (IQR). 3 month old and 18 month old groups had 4 mice; 27 month old group had 3 mice. Since it was impossible to apply normality test for such small groups, nonparametric Mann-Whitney Test (one tail) was used to analyze statistical significance of the differences between the groups. P<0.05 was considered to indicate a significant difference.
